# Phase separation in plants: mechanisms, functions, and a simplified workflow for investigation

**DOI:** 10.3389/fpls.2026.1858323

**Published:** 2026-06-08

**Authors:** Hai Shen, Xue Zou, Peihua Li, Xueli Huang, Shunlin Zheng, Zhitong Ren

**Affiliations:** 1 Panxi Crop Improvement Key Laboratory of Sichuan Province, Xichang University, Xichang, China; 2 College of Agronomy, Sichuan Agricultural University, Chengdu, China; 3 Crop Germplasm Innovation and Genetic Improvement Key Laboratory of Sichuan Province, Mianyang Academy of Agricultural Sciences, Mianyang, China; 4 State Key Laboratory of Crop Gene Exploration and Utilization in Southwest China, Sichuan Agricultural University, Chengdu, China

**Keywords:** functions, liquid-liquid phase separation (LLPS), mechanisms, plant research, workflow

## Abstract

Liquid-liquid phase separation (LLPS) has emerged as a fundamental mechanism organizing membrane-less compartments within cells, driving crucial biological processes. In plants, LLPS facilitates the spatiotemporal regulation of diverse functions, from growth and development, such as hormone signaling, photo-perception, and floral transition, to adaptive responses against abiotic and biotic stresses. This review outlines the properties and major driving forces governing LLPS, emphasizing multivalent interactions mediated by intrinsically disordered regions, repeated domains, and nucleic acids. Key influencing factors, including concentration, temperature, and ionic conditions, are discussed. We further describe a streamlined experimental workflow for studying plant LLPS, encompassing prediction, assessment, and validation. Understanding LLPS dynamics offers profound insights into plant adaptation and resilience, positioning phase separation as a pivotal regulatory paradigm in plant biology.

## 1 Introduction

As the fundamental structural and functional units of living organisms, cells contain numerous membrane-bound organelles, such as the nucleus, chloroplasts, mitochondria, and the endoplasmic reticulum (Xu et al., 2021; Cuevas-Velazquez and Dinneny, 2018). These organelles form relatively independent compartments within the cell, providing specific sites for biochemical reactions and executing unique physiological functions (Emenecker et al., 2020). Concurrently, studies have shown that cells also harbor a substantial number of membrane-less organelles, such as the nucleolus, stress granules, P-bodies, Cajal bodies, DNA damage bodies and so on (Banani et al., 2017; Alberti et al., 2018; Emenecker et al., 2020). These Membrane-less organelles, also referred to as biomolecular condensates, can likewise form distinct compartments within cells and play crucial roles in numerous biological processes (Weber and Brangwynne, 2012; Mitrea et al., 2022; Emenecker et al., 2021).

Phase separation has emerged as a frontier and focal point in life sciences research in recent years. This biophysical process describes the spontaneous separation of a supersaturated solution into two coexisting phases: one concentrated and one dilute, both thermodynamically stable (Boeynaems et al., 2018). Based on the properties of the formed condensates, phase separation can be categorized into liquid-liquid phase separation (LLPS) and liquid-solid phase separation, with LLPS being the most common type in biological systems. LLPS is a thermodynamic process in which a homogeneous solution of macromolecules spontaneously demixes into two coexisting liquid phases: a condensed phase enriched in the solute, and a dilute phase depleted of the solute (Alberti, 2017; Banani et al., 2017; Boeynaems et al., 2018; Cuevas-Velazquez and Dinneny, 2018; Alberti et al., 2019; Gomes and Shorter, 2019; Snead and Gladfelter, 2019; Emenecker et al., 2020; Vendruscolo and Fuxreiter, 2023). In *Caenorhabditis elegans*, P-body collision and fusion were observed, suggesting that LLPS is a key mechanism underlying membrane-less organelle formation (Brangwynne et al., 2009). Upon phase separation, the system enters a two-phase regime in which a dense phase enriched in protein coexists with a dilute phase. The protein concentration in the dense phase remains at the saturation concentration, defining a stable thermodynamic equilibrium. This membrane-less compartment permits macromolecular diffusion and dynamic exchange with the surrounding dilute phase. When the total protein concentration falls below the saturation concentration ​, the dense phase dissolves and the system recover to a homogeneous one-phase state. The phase transition is a highly dynamic and reversible process. A growing body of evidence suggests that phase separation may be a widespread biological phenomenon, playing vital roles in a series of physiological and biochemical processes including biomolecular organization (Feric et al., 2016), biomolecular localization (Banani et al., 2017), environmental stress response, transcriptional regulation (Boija et al., 2018), growth and development (Dong et al., 2026), RNA splicing and processing (Fan et al., 2024), DNA repair (Zhao et al., 2025), and signal transduction (Li et al., 2025). However, current research on phase separation in plants has largely focused on the functions of phase-separating proteins and their roles in biological processes. Systematic summaries of standard procedures and standardized workflows for plant phase separation studies remain scarce. In this study, we aim to provide a simple and practical workflow that can serve as a guideline for future phase separation research.

## 2 Properties of liquid-liquid phase separation

Biomolecular condensates formed via LLPS display a set of characteristic biophysical properties, although no single feature is definitive (Alberti et al., 2019; Li et al., 2012). First, condensates often exhibit a spherical morphology, which minimizes surface tension, but this shape alone does not distinguish liquid-like condensates from other membrane-less compartments, nor is it universal across all material states (Hyman et al., 2014; Emenecker et al., 2021). Second, internal components of condensates are mobile, allowing dynamic exchange with the surrounding dilute phase. Fluorescence recovery after photobleaching (FRAP) is commonly used to assess this mobility: a rapid and substantial recovery indicates liquid-like behavior, whereas slow or no recovery suggests a more solid-like or gel-like state. However, partial recovery can also occur in some non-liquid assemblies, so FRAP alone is insufficient as a standalone criterion (Alberti et al., 2019; Brangwynne et al., 2011; McSwiggen et al., 2019). Third, liquid-like condensates tend to fuse upon contact and relax into a spherical shape, reflecting their fluidity and surface tension. Nevertheless, many functional condensates either do not fuse or undergo maturation into less dynamic states over time (Patel et al., 2015; Woodruff et al., 2018). Therefore, unambiguous demonstration of LLPS typically requires a combination of multiple lines of evidence, including *in vivo* imaging (FRAP, fusion assays), *in vitro* reconstitution with purified components, and sensitivity to 1,6-hexanediol (Alberti et al., 2019; Emenecker et al., 2021; Gao et al., 2022).

## 3 Major drivers of LLPS

The formation of biomolecular condensates is a complex process driven primarily by multivalent interactions among macromolecules. Proteins often contain multiple modular domains arranged in tandem, which can engage in multivalent interactions through their respective binding interfaces. The most common drivers of phase separation are intrinsically disordered regions (IDRs) or low-complexity domains (LCDs) (Cuevas-Velazquez and Dinneny, 2018). IDRs are defined by their conformational flexibility and lack of stable tertiary structure, whereas LCDs are characterized by repetitive, residue-biased sequences (Han et al., 2012; Kato et al., 2012). The conformational plasticity of IDRs and the multivalent interactions mediated by LCDs collectively constitute the structural basis for driving phase separation. For instance, methylation of arginine weakens its interaction with the aromatic ring of tyrosine, thereby reducing the phase separation propensity of FUS protein (Burke et al., 2015). An important type of LCD which is named prion-like domains (PLDs), enriches in uncharged polar amino acids and punctuates by aromatic residues, facilitates the formation of regions with a potential for self-assembly and aggregation (Malinovska et al., 2015; Jung et al., 2020). A second mechanism involves phase separation mediated by specific interactions among multiple repeated domains, where the flexibility between repeats provides the dynamics for interaction. Examples include linear tandem repeats of identical interaction domains, as demonstrated by engineered proteins consisting of multiple SH3 domains or proline-rich motifs (PRMs) (Li et al., 2012). Additionally, protein oligomerization can promote phase separation by increasing interaction valency and local concentration, as exemplified by nucleophosmin (NPM1), which forms pentamers via its N-terminal domain (Vieregg et al., 2018). A third driver is nucleic acid-mediated phase separation, where the flexibility of single-stranded DNA or RNA provides dynamic interaction platforms. Studies have shown that single-stranded nucleic acids are more prone to aggregation and droplet formation than double-stranded ones (Fromm et al., 2014). The diverse intramolecular and intermolecular multivalent interactions established among these biomolecules provide the intrinsic driving force for phase separation and contribute to the complexity and variability of their molecular composition and structural basis for phase separation.

## 4 Factors influencing LLPS formation

Phase separation is a dynamic process influenced by multiple factors, regulated not only by driving forces but also closely associated with various environmental stimuli. Generally, at low concentrations, biomolecules remain uniformly dispersed in the solvent. Only upon exceeding a critical saturation concentration do attractive interactions lead to condensation and phase separation. Thus, a threshold concentration is required for macromolecules to undergo phase separation. Furthermore, temperature and salt concentration also affect these processes. The influence of temperature reflects entropic effects: since phase separation reduces system entropy, elevated temperatures typically hinder phase transition for most biomolecules (Brady et al., 2017). However, for some heat shock-related proteins, increased temperature can promote phase separation (Tong et al., 2022). Electrostatic interactions mediated by charged residues can promote phase separation, whereas increased salt concentration screens these interactions and thus suppresses phase separation (Brady et al., 2017). In specific contexts, metal ions may modulate or bridge electrostatic interactions, but they are not the primary drivers. Conversely, if hydrophobic interactions dominate the intermolecular forces, increased salt concentration may promote phase separation, as observed with the RNA-binding protein FUS (Burke et al., 2015). pH conditions can alter the physicochemical properties of amino acids, thereby influencing phase separation propensity under specific environmental conditions (Franzmann et al., 2018). In addition, macromolecular crowding promotes protein aggregation and phase separation by elevating local protein concentrations, which enhances productive collisions and effective intermolecular interactions. Lowering the critical concentration threshold, crowding thus drives phase separation and assembly (Holt and Delarue, 2023).

## 5 Biological functions of LLPS in plants

**Figure 1:**
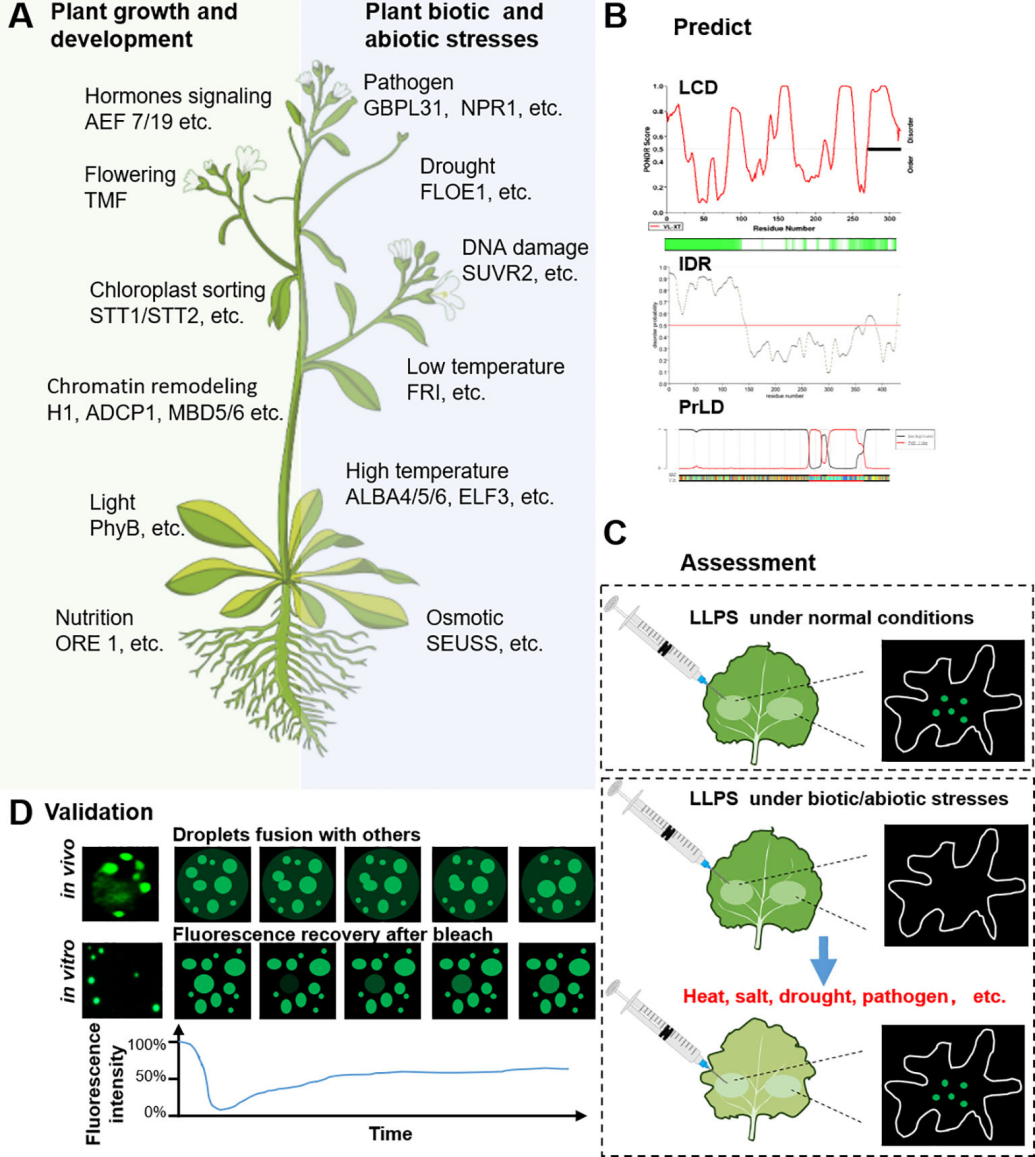
Biological function of LLPS and a simplified workflow for studying LLPS in plants. **(A)** Biological function of LLPS in plant growth, development and biotic/abiotic stresses. **(B)** Prediction programs for IDRs/LCDs/PrLDs of target proteins. **(C)** Transient experiment to evaluate the potential of phase separation in tobacco. **(D)** To validate the LLPS of target protein, a series of experiments (*in vivo* and *in vitro*) to assess the LLPS phenomenon and its biological properties. The vectors constructed by deleting the IDR or replacing it with the IDR of other proteins can be used for genetic verification.

Membrane-less organelles formed via biomolecular phase separation can recruit and enrich functional effector molecules involved in specific biological processes, enabling the spatial compartmentalization of relevant macromolecules and participating in the catalysis and efficiency regulation of these processes (Snead and Gladfelter, 2019; Dragwidge and Van Damme, 2023). On the other hand, the process of phase separation facilitates the formation of distinct intracellular compartments, contributing to the regulation of specific cellular biological processes. Current research on biomolecular phase separation has primarily focused on animal and yeast cells, linking it to DNA organization, damage repair, RNA transcription and translation, as well as cell proliferation, aging, signal transduction, cell cycle regulation, stress protection, and disease pathogenesis (Fan et al., 2024). Recent studies indicate that phase separation is also involved in plant growth (Liu et al., 2022), development (Ouyang et al., 2020), RNA-binding process (Fan et al., 2024) immune responses (Zavaliev et al., 2020), heat stress response (Tong et al., 2022), gradually becoming a hotspot in plant research (**Figure 1A**) (Cuevas-Velazquez and Dinneny, 2018; Emenecker et al., 2020; Xu et al., 2021; Wang et al., 2022; Vendruscolo and Fuxreiter, 2023; Liu et al., 2024).

### 5.1 Biological functions in plant growth and development

LLPS participates in regulating plant growth and development, including hormone signal transduction, floral transition, light perception, and chloroplast sorting (Emenecker et al., 2020; Xu et al., 2021; Fan et al., 2024). Although hormones are central to plant growth and development, phase separation has been documented only for certain hormonal pathways, where it contributes to the regulation of gene expression and related metabolic reactions (Emenecker et al., 2020). For example, ARF7 and ARF19, two activating ARF transcription factors, form cytoplasmic condensates via their low-complexity middle region and PB1 domain. In actively growing tissues, they localize to the nucleus and promote auxin-responsive gene expression, whereas in mature tissues they are sequestered in cytoplasmic condensates, which reduces their nuclear activity and attenuates auxin responses (Korasick et al., 2015; Powers et al., 2019). The strigolactone signaling repressor SMXL7 undergoes phase separation to form nuclear condensates, where it recruits signaling components and transcription factors to repress gene transcription in *Arabidopsis* (Li et al., 2025) Phase separation can also mediate plant senescence under nutrient deficiency. For instance, MED19a interacts with the senescence-associated transcription factor ORE1 in a phase-separated manner, leading to leaf yellowing and senescence under nitrogen-deficient conditions (Cheng et al., 2022). Phytochromes are the primary photoreceptors in plants, involved in photomorphogenesis and seed germination. Phytochrome B (PhyB) contains an N-terminal IDR and a C-terminal region with strong oligomerization capacity. These structural features enable self-aggregation and reversible, light-controlled conformational changes coupled with temperature-dependent phase separation, allowing plants to perceive both light and temperature cues (Chen et al., 2022). The flowering process is influenced by multiple factors, where reactive oxygen species (ROS) can act as signaling molecules. The transcription factor TMF (Terminating Flower), a key regulator of tomato shoot apical meristem development, responds to ROS signals by forming disulfide bonds between TMF proteins, promoting the formation of large condensates that suppress the expression of the florigen gene *AN* (Huang et al., 2021b). In addition to controlling flowering, floral organ abscission is also closely linked to phase separation. Phase separation of BLADE-ON-PETIOLE (BOP) transcriptional cofactors, together with the transcription factor TMF FAMILY MEMBER 1 (TFAM1), promotes abscission zone formation and orchestrates programmed petal abscission and senescence in tomato (Xiao et al., 2026). Beyond light perception and hormone signaling, phase separation also mediates the perception of temperature signals in plant developmental processes. In *Arabidopsis*, the thermosensor ELF3 undergoes phase separation via its prion-like domain (PrD) in response to high temperature, thereby releasing from chromatin and alleviating transcriptional repression (Jung et al., 2020). Phase separation also acts as a negative regulator of transcription factors in the case of FRIGIDA (FRI) condensates in the nucleus, thereby preventing FRI from activating *FLC*, and allowing plants to sense seasonal progression and regulate flowering time (Zhu et al., 2021).Chromatin remodeling, as an important epigenetic mechanism, plays a crucial role in regulating various developmental processes in plants, such as embryonic development, seed germination, flowering, and photomorphogenesis (Liu et al., 2024). The phase separation of some important proteins plays a crucial role in the process of chromatin remodeling, such as the linker protein H1, agenet domain containing protein 1 (ADCP1), histone modifications related proteins, MORC proteins and so on (Gao et al., 2019; He et al., 2019; Ries et al., 2019; Zhao et al., 2019; Fu and Zhuang, 2020; Buttress et al., 2022; Wang et al., 2023; He et al., 2024; Lee et al., 2024). Methyl-CpG-binding domain protein (MBD5) and MBD 6, and two α-crystallin domain–containing proteins (ACD 15.5 and ACD21.4) form a complex, which recruits MORC6 to regulate selective expression of rRNA gene variants (Ren et al., 2024).

### 5.2 Biological functions in stresses

Plants also employ phase separation to cope with various abiotic and biotic stresses, such as pathogen infection, drought, salinity, and extreme temperatures that cause cellular damage (Franzmann and Alberti, 2019; Emenecker et al., 2020; Xu et al., 2021; Fan et al., 2024). Liquid–liquid phase separation has emerged as a key mechanism that organizes signaling and stress responses, playing an essential role in plant adaptation to a wide range of environmental stresses. Green evolution-associated DELLA accumulation enhances salt tolerance in cereals by disrupting the phase separation of the transcriptional repressor INDETERMINATE SPIKELET1 (IDS1), thereby reprogramming sugar-amino acid metabolic networks (Cheng et al., 2026). Phase separation of the *Arabidopsis* DCP5 protein, driven by hyperosmotically intensified molecular crowding and sensed by its intrinsically disordered region, assembles cytoplasmic osmotic stress granules that sequester mRNAs and regulatory proteins to reprogram translatome and transcriptome for stress adaptation (Wang et al., 2024). Phase separation of the transcriptional regulator SEUSS, driven by hyperosmolarity-induced molecular crowding, promotes stress tolerance gene expression in *Arabidopsis* (Wang et al., 2022) Phase separation of sucrose nonfermenting 1–related protein kinase 2 (SnRK2), triggered by molecular crowding under severe osmotic stress, disrupts ABI1-mediated inhibition to activate osmotic stress signaling (Yuan and Zhao, 2025). FLOE1 phase separates in response to hydration and acts as a water sensor. Under drought stress, FLOE1 stays diffuse and inhibits germination; upon rehydration, condensate formation alleviates this inhibition and promotes germinationGreen (Dorone et al., 2021). Plants also can utilize phase separation to modulate tolerance to heat and cold stress. Heat-induced phase separation of FUST1, mediated by a prion-like domain-encoded thermoswitch, primes stress granule assembly and promotes thermotolerance in *Arabidopsis* (Geng et al., 2025). The RNA-binding protein ALBA undergoes heat-induced phase separation tand localizes to stress granules and processing bodies, where it directly binds and sequesters heat stress-related mRNAs (such as HSF mRNAs), thereby protecting them from XRN4-mediated degradation and enhancing thermotolerance (Tong et al., 2022). RBGD2/4 proteins, via tyrosine-rich repeats (TRA) within their LCDs, drive LLPS and interact with multiple stress granule components (e.g., PAB2/4/8), protecting plants from heat damage (Zhu et al., 2022). Heat-induced solid-like condensation of MORF8 suppresses chloroplast RNA editing by sequestering PPR proteins, leading to impaired NDH activity and reduced photosynthesis (Wu et al., 2025). Under cold stress, CBF transcription factors accumulate and interact with the spliceosome component SKIP to promote its nuclear condensation, thereby enhancing the alternative splicing efficiency of cold-responsive transcripts and positively regulating cold acclimation (Fu et al., 2025). Upon encountering biological stress, the formation of LLPS regulates the transcription of disease-resistance genes and bolsters plant immunity. In *Arabidopsis*, guanylate-binding protein-like GTPases (GBPLs) undergo phase separation upon salicylic acid treatment or pathogen infection. Immune signals trigger GBPL3 phase separation, forming immune condensates (GDACs) that significantly enhance the transcription of defense genes and boost plant immunity (Huang et al., 2021a). Fusarium head blight (FHB) is one of the most serious wheat diseases worldwide. While TaHRC (Histidine-rich calcium-binding protein) alleles oppositely regulate condensation of a multi-IDR protein hub, which in turn differentially controls global alternative splicing to determine wheat susceptibility or resistance to Fusarium head blight (He et al., 2024). The tomato SR30 splicing factor undergoes phase separation to repress alternative splicing of defense-related genes, thereby negatively regulating immunity against oomycete pathogens (Yan et al., 2025).

## 6 A simplified workflow for studying LLPS in plants

As an emerging field in plant biology and functional research, a simplified, generalizable experimental workflow for LLPS is essential. This typically involves three main steps: prediction, assessment, and validation (**Figure 1**).

### 6.1 Prediction

To identify potential plant proteins capable of phase separation, the initial step is to predict whether the target protein possesses specific domains such as IDRs, LCDs, or PrLDs. A variety of prediction tools and web servers have been developed for this purpose. For example, platforms like FuDrop (https://fuzdrop.bio.unipd.it) (Hatos et al., 2022) and dSCOPE (http://dscope.omicsbio.info) (Yu et al., 2023) are used for comprehensive analysis; LLPSDB (http://bio-comp.org.cn/llpsdb/home.html) (Li et al., 2020a) and PhaSePro (https://phasepro.elte.hu/) (Mészáros et al., 2020) are regarded as integrated database; while D^2^P^2^ (http://d2p2.pro/) (Oates et al., 2012), Pondr (http://pondr.com/), PrDOS (http://prdos.hgc.jp) (Ishida and Kinoshita, 2007), IUPred3 (https://iupred.elte.hu) (Erdős et al., 2021) and PLAAC (http://plaac.wi.mit.edu) (Lancaster et al., 2014) can predict these IDR or PrLDs features (**Figure 1B**). However, these tools differ fundamentally: sequence-based predictors (e.g., IUPred3, PrDOS) estimate disorder propensity, whereas motif-based tools (e.g., PLAAC) identify prion-like amino acid composition. None directly measure phase separation. Importantly, most LLPS databases embed predictors (e.g., IUPred, LLPSDB, PLAAC) without a standard negative dataset (proteins confirmed not to phase separate), include proteins localized to membraneless organelles without direct LLPS validation, and rely heavily on computational orthologs. Consequently, predictors tend to overestimate phase-separation propensity, especially for intrinsically disordered regions, generating substantial false positives. Therefore, computational prediction serves only as a hypothesis-generating filter; experimental confirmation remains the gold standard (Li et al., 2020b).

### 6.2 Assessment

To further verify the phase separation capability of a target protein, it is common to construct expression vectors driven by its native promoter rather than a strong constitutive promoter (35S), to avoid artificial condensation due to overexpression. Native promoters maintain physiological protein levels, whereas constitutive promoters may cause false positives by exceeding the saturation concentration. Transient expression in model systems such as tobacco (*Nicotiana benthamiana*) allows rapid visualization. Compared with the GFP control, the target protein can form large molecular condensates. Some proteins may require specific treatments (such as heat, salt or osmotic stress) to induce condensate formation (**Figure 1C**). A critical control is treatment with 1,6−hexanediol, which disrupts hydrophobic interactions and reversibly melts many phase−separated condensates; sensitivity to 1,6−hexanediol supports liquidity but is not definitive (Zheng et al., 2025).

### 6.3 Validation

Once transient expression confirms the protein’s phase separation ability, a series of experiments is required to validate its characteristics (**Figure 1D**).

*In vivo* validation: (i) FRAP a recovery half-time from seconds to minutes indicates liquid-like dynamics (Ren et al., 2024); (ii) droplet fusion–adjacent condensates coalesce; (iii) 1,6-hexanediol sensitivity–supports liquidity but is not definitive (Zheng et al., 2025; Ouyang et al., 2020); (iv) domain deletion–removing predicted IDR/LCD/PrLD abolishes condensation (Xie et al., 2021); (v) heterologous IDR replacement–swapping the IDR with that from another species restores both condensation and function, proving that multivalency, not specific sequence, drives LLPS (Dragwidge et al., 2024). *In vitro* validation (performed after *in vivo* to avoid chasing artifacts): (i) purified recombinant protein; (ii) matching physiological concentration–excessively high concentrations may cause false positives; (iii) determination of saturation concentration; (iv).FRAP (Huang et al., 2025), fusion (Tong et al., 2022), sensitivity to salt concentration (Xie et al., 2021), and responses to environmental factors (Cheng et al., 2026). However, it should be noted that the concentration of purified protein used in *in vitro* experiments must match its physiological concentration *in vivo*. Excessively high *in vitro* concentrations may produce false positives that do not necessarily represent genuine phase separation under native physiological conditions.

Genetic analysis: CRISPR-based in-frame deletion of the IDR in the endogenous locus is preferred over transgenic overexpression. Criteria for functional LLPS: converging evidence from (i) *in vivo* liquidity (FRAP, fusion, 1,6-HD sensitivity), (ii) *in vitro* reconstitution at physiological, (iii) loss-of-condensation mutations that ablate biological function, and (iv) heterologous IDR rescue that restores both condensation and function (Xie et al., 2021).

In summary, a robust demonstration of biologically relevant phase separation requires converging evidence from multiple orthogonal techniques: (1) *in vivo* liquid-like behavior (FRAP recovery, fusion, and 1,6-hexanediol sensitivity), (2) *in vitro* reconstitution at physiological concentrations, (3) loss-of-phase-separation mutations that ablate function, and (4) heterologous IDR replacement that restores both condensation and function. No single experiment is sufficient; the field increasingly accepts that a combination of these criteria provides confidence in assigning functional relevance to LLPS.

## 7 Summary and outlook

Phase separation is an emerging research paradigm playing an increasingly important role in elucidating biomolecular functions, yet several challenges remain. Most plant studies rely on overexpressed fluorescent fusions, which may overestimate condensation propensity; endogenous labeling (such as CRISPR and knock-in) and monomeric fluorophores are urgently needed. Moreover, the native composition and material properties of condensates are poorly understood. Proximity labeling combined with crosslinking, and super-resolution microscopy (STED), should be applied to capture transient components and sub-200 nm condensates. Manipulating phase separation without disrupting intrinsic protein function is difficult. Heterologous IDR swapping and mutation of sequence (charge, aromatic residues) offer promising strategies. Another major need is to move beyond Arabidopsis to crops. Natural IDR variations provide allelic series that tune phase behavior and can be exploited for breeding. Large-scale identification of crop LLPS proteins is required, but current predictors suffer high false-positive rates due to lack of reliable negative datasets, so a simple and practical workflow is essential. Emerging trends include integrating phase separation with post-translational modifications, RNA modifications (m^6^A), and metabolite signals. Synthetic condensates via designed IDRs or engineered scaffolds hold promise for programmable subcellular compartmentalization. Future research should prioritize rigorous *in vivo* validation, dissection of condensate composition, functional manipulation via IDR replacement, translation to crops using natural variation and editing, and integration with signaling networks. A standardized workflow combining live imaging, proximity labeling, reconstitution, and computational prediction will greatly facilitate progress.
